# Genotype‐function‐phenotype correlations for *SCN1A* variants identified by clinical genetic testing

**DOI:** 10.1002/acn3.52297

**Published:** 2025-01-21

**Authors:** Andrew T. Knox, Christopher H. Thompson, Dillon Scott, Tatiana V. Abramova, Bethany Stieve, Abigail Freeman, Alfred L. George

**Affiliations:** ^1^ Department of Neurology University of Wisconsin School of Medicine and Public Health Madison Wisconsin USA; ^2^ Department of Pharmacology Northwestern University Feinberg School of Medicine Chicago Illinois USA

## Abstract

**Objective:**

Interpretation of clinical genetic testing, which identifies a potential genetic etiology in 25% of children with epilepsy, is limited by variants of uncertain significance. Understanding functional consequences of variants can help distinguish pathogenic from benign alleles. We combined automated patch clamp recording with neurophysiological simulations to discern genotype‐function‐phenotype correlations in a real‐world cohort of children with *SCN1A*‐associated epilepsy.

**Methods:**

Clinical data were extracted for children with *SCN1A* variants identified by clinical genetic testing. Functional properties of non‐truncating Na_V_1.1 variant channels were determined using automated patch clamp recording. Functional data were incorporated into a parvalbumin‐positive (PV+) interneuron computer model to predict variant effects on neuron firing and were compared with longitudinal clinical data describing epilepsy types, neurocognitive outcomes, and medication response.

**Results:**

Twelve *SCN1A* variants were identified (nine non‐truncating). Six non‐truncating variants exhibited no measurable sodium current in heterologous cells consistent with complete loss of function (LoF). Two variants caused either partial LoF (L479P) or a mixture of gain and loss of function (I1356M). The remaining non‐truncating variant (T1250M) exhibited normal function. Functional data changed classification of pathogenicity for six variants. Complete LoF variants were universally associated with seizure onset before one year of age and febrile seizures, and were often associated with drug resistant epilepsy and below average cognitive outcomes. Simulations demonstrated abnormal firing in heterozygous model neurons containing dysfunctional variants.

**Interpretation:**

In *SCN1A*‐associated epilepsy, functional analysis and neuron simulation studies resolved variants of uncertain significance and correlated with aspects of phenotype and medication response.

## Introduction

Epilepsy is a common childhood disorder, and one third of children with epilepsy have lower quality of life due to drug‐resistant seizures.^1^, ^2^ Greater availability of clinical genetic testing has the potential to reduce the prevalence of drug‐resistant epilepsy by guiding more precise therapy. Genetic testing can identify potentially causal variants at seizure onset in 25% of children with epilepsy.^3^, ^4^, ^5^ By providing a specific etiology, genetic testing can help limit unnecessary diagnostic workup, predict disease course, and identify gene‐specific treatments at time of diagnosis. However, genetic variants do not exist in a vacuum; epilepsy is a network disorder, and other developmental, structural, or genetic factors can contribute to substantial phenotypic variability, even for affected individuals with the same variant.^6^, ^7^, ^8^, ^9^ Additionally, variants of uncertain significance (VUS) are common,^10^, ^11^ and together with variable phenotype/genotype correlations limit clinicians' ability to establish genetic diagnoses and utilize precision medicine approaches.

Pathogenic *SCN1A* variants are among the most common identified etiology for monogenic epilepsy,^4^, ^5^, ^12^ and are associated with a variety of epilepsy phenotypes, ranging from mild (genetic epilepsy with febrile seizures plus [GEFS+]) to severe (Dravet syndrome).^13^, ^14^ *SCN1A* encodes a voltage‐gated sodium channel (Na_V_1.1) expressed predominantly in parvalbumin‐positive (PV^+^) interneurons.^15^ The effects of a genetic variant on Na_V_1.1 function may be measured using voltage‐clamp recording of heterologously expressed recombinant channels, and effects on neuronal firing can be predicted using computational neuron simulations.^16^, ^17^ Functional analysis of Na_V_1.1 VUS can provide information useful for assessment of pathogenicity and the pattern of channel dysfunction can correlate with clinical phenotype.^18^, ^19^ Furthermore, detailed determination of genotype–function–phenotype correlation is potentially useful for optimizing therapy in *SCN1A*‐related epilepsy.

Previous studies of genotype‐phenotype correlations for *SCN1A* variants have not systematically included functional assessments and do not provide a representative cross section of *SCN1A* variants identified by present day genetic testing paradigms utilized in individuals with new onset epilepsy.^12^, ^18^, ^19^, ^20^ To better understand the spectrum of genotype‐function‐phenotype correlations for individuals with epilepsy and *SCN1A* variants identified by clinical genetic testing, we used automated voltage clamp recording to determine the functional effects of epilepsy‐associated *SCN1A* channel variants identified in a tertiary care center. We supplemented functional testing with computer simulations to predict the effects of channel dysfunction on PV^+^ interneuron firing, and correlated these data with a detailed, longitudinal description of clinical course and antiseizure medication (ASM) response.

## Methods

### Study participants and clinical data

This study was approved by the institutional review board of the University of Wisconsin–Madison. We included all individuals with epilepsy who had an exonic *SCN1A* variant identified by clinical genetic testing before January 2021 who were encountered in the pediatric neurology clinic between January 2018 and December 2022. Individuals were identified by (1) querying electronic medical records for patients with ICD codes for Dravet syndrome or genetic epilepsy with febrile seizures+ (GEFS+), (2) querying an institutional database of patients with epilepsy, and (3) querying an institutional epilepsy gene panel test database. Seizure semiology, frequency, duration, and severity, as well as medication dates, dosages, and patient weights were extracted from medical records. ASM doses were normalized with “Full Dose” representing a typical maximal dose in mg/kg/day (levetiracetam: 80, zonisamide: 10, phenobarbital: 6.0, valproic acid: 40, clobazam: 1.0, epidiolex: 20, clonazepam: 0.2, fenfluramine: 0.7, topiramate: 10, perampanel: 0.3, lamotragine: 8.0, brivaracetam: 1.5, oxcarbazepine: 45). Treatment with ketogenic diet or vagal nerve stimulator at any level were represented as “Full Dose.” Nine of eleven study participants continued care at University of Wisconsin at the end of the study period; one moved out of state, and one individual died of SUDEP.

### Plasmids and mutagenesis

Full‐length cDNAs encoding intron‐stabilized human WT or variant Na_V_1.1 corresponding to the adult splice isoform (NCBI accession NM_001165963)^21^ were engineered into plasmid vectors with an internal ribosome entry site (IRES) followed by the monomeric red fluorescent protein mScarlet (pIRES2‐mScarlet) as previously described.^16^ Variants were introduced into Na_V_1.1 by site‐directed mutagenesis using Q5 2X high‐fidelity DNA polymerase Master Mix (New England Biolabs, Ipswich, MA) as previously described.^21^ Mutagenic primer sequences are provided in Table S1.

### Functional evaluation of SCN1A variants

For automated patch clamp recording, WT or variant Na_V_1.1 cDNAs were electroporated into HEK293T cells using the MaxCyte STX electroporation system (MaxCyte Inc., Gaithersburg, MD, USA) using methods described previously for Na_V_1.2.^16^ HEK293T cells were maintained in Dulbecco's modified Eagle's medium (GIBCO/Invitrogen, San Diego, CA, USA) supplemented with 10% fetal bovine serum (Atlanta Biologicals, Norcross, GA, USA), 2 mM l‐glutamine, 50 units/mL penicillin, and 50 mg/mL streptomycin at 37°C in 5% CO_2_.

Automated patch clamp recording was performed as previously described for Na_V_1.2,^16^ using the Nanion Syncropatch 768PE platform (Nanion Technologies, Munich, Germany) using single‐hole thin‐wall Type‐S resistance (2–3 MΩ) recording chips. Pulse generation and data collection were performed using PatchControl384 v1.9.1 and DataControl384 v1.9.0 software (Nanion Technologies). Whole‐cell currents were acquired at 10 kHz, series resistance was compensated 80%, and leak currents were subtracted using P/4 subtraction. The external solution contained (in mM): 140 NaCl, 4 KCl, 2 CaCl_2_, 1 MgCl_2_, 1 HEPES, 5 glucose, with the final pH adjusted to 7.4 with NaOH, and osmolality adjusted to 300 mOsm/kg/L with sucrose. The composition of the internal solution was (in mM): 110 CsF, 10 CsCl, 10 NaCl, 20 EGTA, 10 HEPES, with the final pH adjusted to 7.2 with CsOH, and osmolality adjusted to 300 mOsm/kg/L with sucrose. High resistance seals were obtained by addition of seal enhancer solutions, which contained (in mM): 80 NaCl, 3 KCl, 35 CaCl_2_, 10 MgCl_2_, 10 HEPES, with the final pH adjusted to 7.4 with NaOH., Cells were washed twice with external solution prior to recording, and the final concentrations of CaCl_2_ and MgCl_2_ were 3 and 2 mM, respectively. Criteria for cell selection were previously described.^16^

Data were analyzed and plotted using a combination of DataControl384 v1.9.0 (Nanion Technologies), Clampfit 10.4 (Molecular Devices), Microsoft Excel (Microsoft Office 2019), and GraphPad Prism (GraphPad Software, San Deigo, CA, USA). Whole‐cell currents were normalized to membrane capacitance. Window current was calculated using a custom MatLab Script (cite: PMID:34287911). One‐way ANOVA with Dunn's post‐hoc test was used for statistical comparison, and the threshold for statistical significance was *p* ≤ 0.05. All data are reported as mean ± 95% confidence interval (95% CI).

### Neuronal action potential simulations

Simulations were carried out in NetPyNE,^22^ a package based on Neuron^23^ and Python.^24^ The multicompartmental PV^+^ interneuron model adapted by Bereki et al. from the Blue Brain Project Microcircuit Portal^17^, ^25^ was ported to this environment and is available at (will make available on modelDB upon acceptance for publication). This model contains a detailed topology with 56 dendritic compartments, a soma, and a simplified axon with a proximal and distal segment. Each compartment contains 11 voltage‐dependent or calcium‐dependent channels, with appropriate representations of channel conductance in different regions. Na_V_1.1 channels are represented using the Hodgkin‐Huxley formalism:

$$I={\mathrm{gNa}}_{V}1.1\times m^{3}\times h\times \left(V-E_{\mathrm{Na}}\right),$$

where *E*_Na_ = 50 mV, *m* and *h* are sodium channel activation and inactivation probabilities, and gNa_V_1.1 is peak channel conductance. Heterozygous genotypes were modeled as two distinct Na_V_1.1 channels in each compartment, each responsible for 50% of total Na_V_1.1 conductance, with variant parameters incorporated into one channel. Percent change in variant Na_V_1.1 conductance and kinetics determined by in vitro experiments were incorporated into the corresponding model parameters for variant Na_V_1.1 channels; variants that resulted in total loss of function were modeled as 0% conductance for the variant channel and 50% conductance for the WT channel to simulate haploinsuffiency. Absolute value of the shift in half‐maximal voltage (*V*_0.5_) of steady state activation or inactivation was modeled by a corresponding absolute shift in *V*_0.5_ of model channel parameters.

We tested the effects of variants on neuronal firing by carrying out runs of multiple 200 ms simulations with varied stimulation parameters. First, we tested the effect of an injected current of varying amplitude at the soma (one run of 66 simulations). We then tested a combination of semi‐regular trains of excitatory post synaptic potentials (EPSPs) and inhibitory post synaptic potentials (IPSPs) at the soma (100 runs of 65–80 simulations). Post synaptic potentials (PSPs) were modeled using a 2‐state kinetic scheme with a rise time constant of 0.01 ms and a decay time constant of 0.5 ms. EPSPs had a reversal potential of 0 mV, IPSPs had a reversal potential of −70 mV; EPSPs and IPSPs had equal amplitude, and stimulation trains were semi‐regular with 20% variability in pulse timing. For each run of simulations, the stimulus amplitude or frequency was titrated based on previous simulation results in phases designed to identify the minimum stimulus that caused the interneuron to fire, stimulus that led to maximal firing rate, and a representative sample of interneuron firing between and beyond these transition points.

### Comparison to in silico prediction tools

To compare the utility of the in vitro and computer modeling studies used here to other in silico prediction tools, we entered variant and clinical data into a publicly available prediction model.^19^ This model takes specific genetic variant and age of epilepsy onset as inputs, and outputs the probability of a Dravet phenotype (vs. a GEFS+ phenotype). We used this model to compute the probability of Dravet syndrome for each individual. To determine the influence of age on model prediction and to compare the ability of the prediction model to distinguish between variants, we tested model output for each variant at a spectrum of ages and compared variant differentiation between the in silico prediction model and our experimental findings.

## Results

### Identification of SCN1A variants

**Figure 1:**
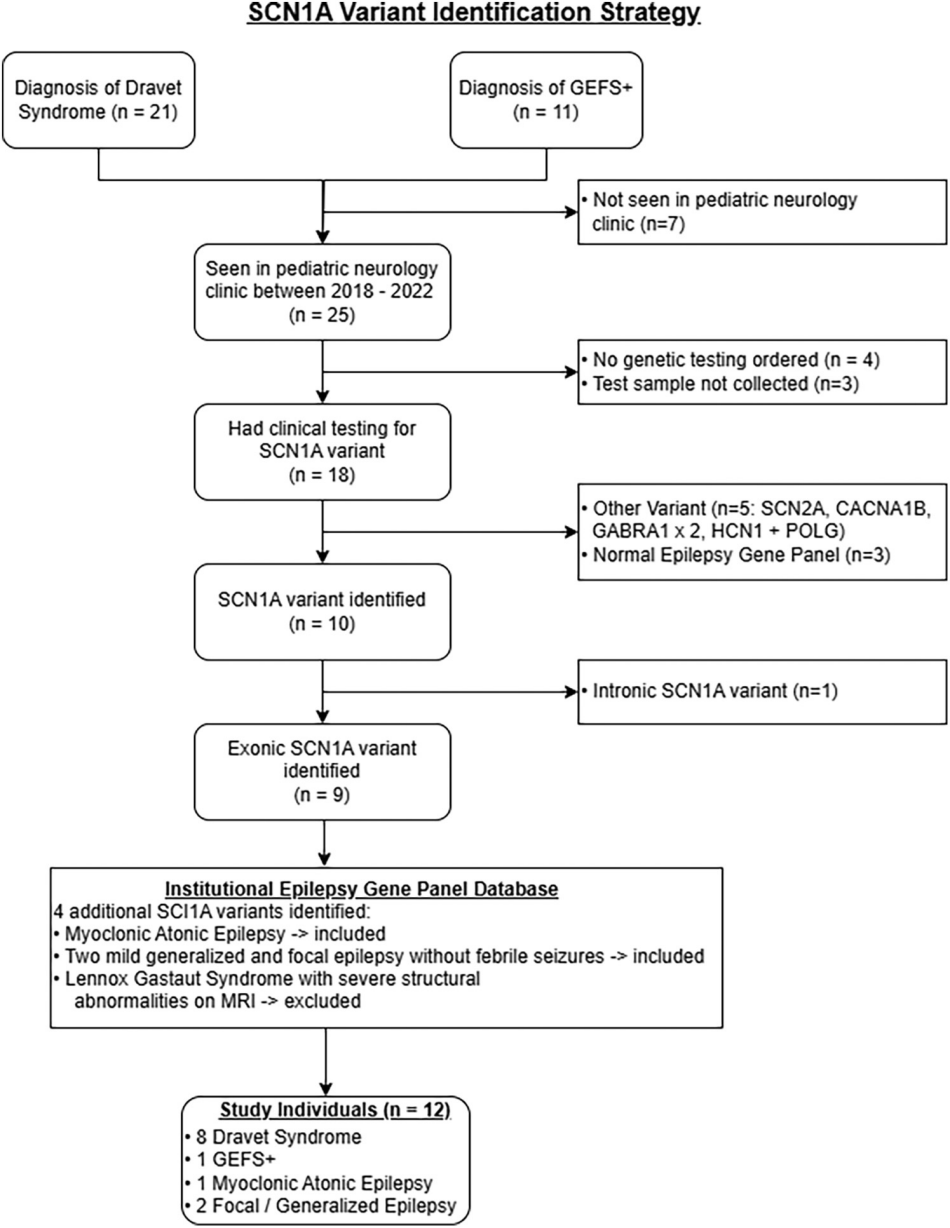
SCN1A variant identification strategy.

**Figure 2:**
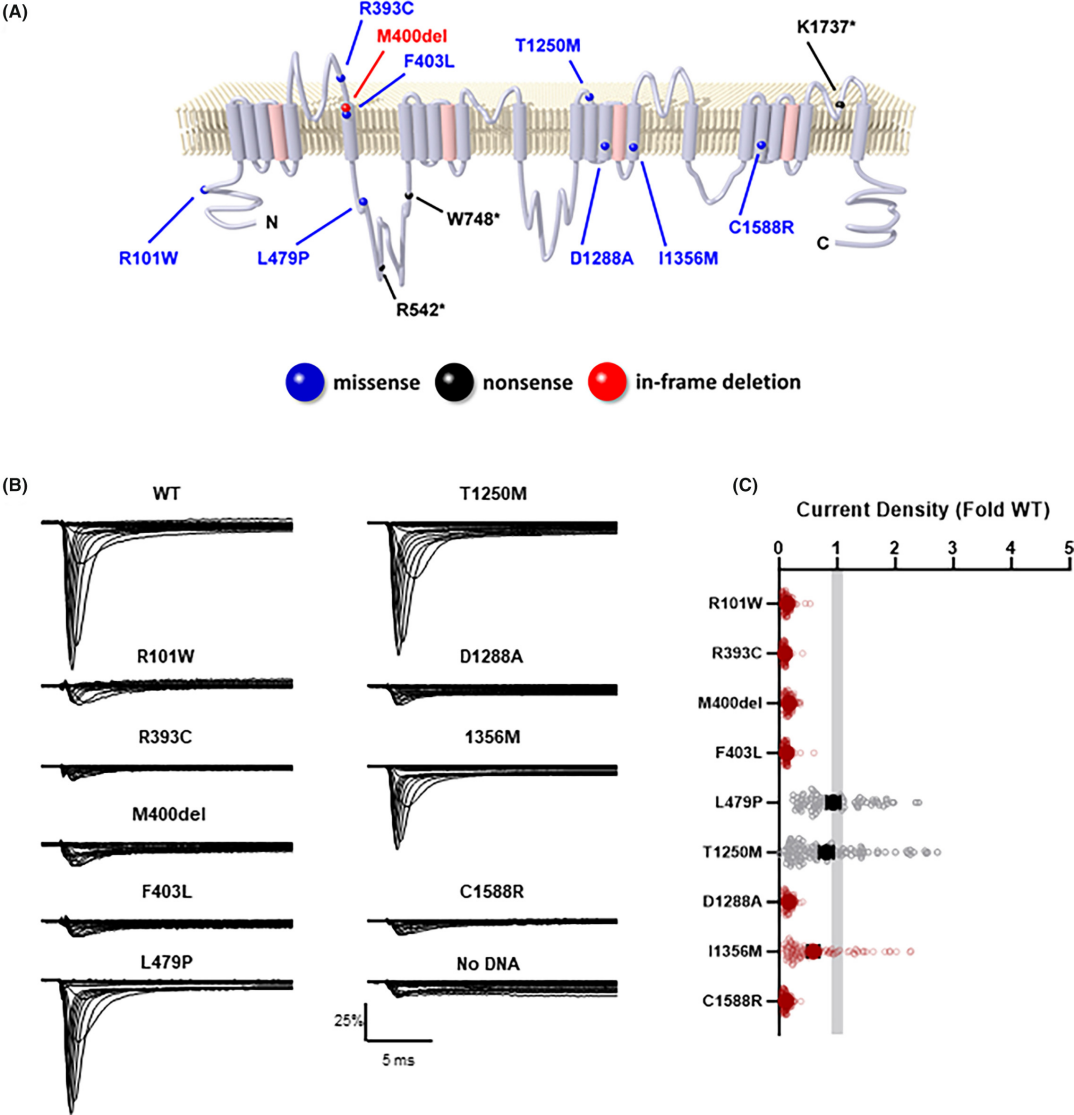
Location and functional properties of SCN1A variants. (A) Locations of variants on a simplified membrane topology of the Na_V_1.1 protein. (B) Average normalized whole‐cell sodium currents for WT and epilepsy‐associated variants (C) Average fold‐deviation of whole‐cell sodium current density from WT SCN1A. All data are plotted as mean ± 95% CI for 78 to 119 cells. The gray bar represents 95% CI for average WT current density. Red symbols denote data that are significantly different from WT (*p* < 0.05).

A review of medical records and an institutional database of patients with epilepsy identified 21 patients with a diagnosis of Dravet syndrome and 11 with a diagnosis of GEFS+ (Fig. 1). Nine of these patients had an exonic *SCN1A* variant identified by clinical genetic testing and were encountered in the pediatric neurology clinic between January 2018 and December 2022. Three additional individuals, one with epilepsy with myoclonic‐atonic seizures and two with generalized and focal epilepsy without febrile seizures, were included after reviewing an institutional database of epilepsy gene panel results, for a final cohort of 12 individuals with epilepsy and heterozygous exonic *SCN1A* variants including three truncating variants (Tables 1 and 2). Truncating variants were not included in functional studies because they were assumed to have complete loss‐of‐function (LoF). The locations of the 12 variants on a simplified membrane topology for the Na_V_1.1 protein are illustrated in Figure 2A.

**Table 1:** Epilepsy phenotypes for individuals with SCN1A variants.

Subject ID	Variant	Age of first seizure	Months followup	Generalized seizure type	Focal seizure type	Seizures >10 min	Status epilepticus	Febrile seizures	# EEGs (normal/abnormal)	EEG abnormality	Epilepsy syndrome	Seizures controlled?	Neuropsych testing age	Full scale IQ	Developmental delay noted	Autism	ADHD
1	I1356M	3 y	3 y	GTC	C‐IA	N	N	N	1/0	–	–	Y	–	–	–	N	Y
2	L479P	5 y	21 m	TA, GTC	C‐IA	N	N	N	1/2	GD, TA	–	Y	8 y	107	–	N	N
3	R101W	5 m	5 y	M	C‐IA, BA	Y	N	Y	1^a^/1	FD, GD, FC, M	DS	Y	9 y	40	18 m	Y	Y
4	K1737*	3 m	15 y	GTC, M	C‐IA	Y	Y	Y	2/3	GD, M, FD	DS	Y	7 y	52	4 y	N	Y
5	C1588R	8 m	5 y	A, GTC, M	C‐PA	N	N	Y	1/1	GD	DS	Y/N^c^	–	–	18 m	N	N
6	M400del	7 m	3 y	GTC, M	C‐PA, T‐PA	Y	N	Y	2/1	FD, M	DS^b^	Y	3 y	85	–	N	N
7	F403L	4 m	19 m	–	C‐IA	Y	Y	Y	1/0	–	FS+	Y	–	–	–	N	N
8	R393C	8 m	27 m	GTC	–	Y	Y	Y	0/2	–	DS	N	–	–	2 y	N	N
9	D1288A	3 m	3 y	GTC	C‐PA	Y	Y	Y	5/1	FD	DS	N	3 y	75	–	Y	B
10	R542*	4 m	29 m	GTC	C‐PA	N	N	Y	1/1	GD	DS	N	–	–	18 m	N	N
11	W748*	11 m	3 y	GTC	C‐IA, BA	Y	N	Y	0/3	FD, GD	DS	N	3 y, 4 y	85, 76	4 y	Y	Y
12	T1250M	2 y	11 y	MA, GTC, AA	–	N	N	N	1/4	FD, GD	EMA	Y	–	–	18 m	N	N

All patients with total loss of function variants had first seizure before one year of age and fevers as a trigger for seizures. Other phenotypic details including seizure types, EEG findings, IQ scores, and presence of developmental delay were variable.

A, atonic; AA, atypical absence; B, borderline ADHD; BA, behavioral arrest; C, clonic; DS, Dravet syndrome; EMA, epilepsy with myoclonic‐atonic seizures; FD, focal epileptiform discharges; FS+, febrile seizures plus; GD, generalized epileptiform discharges; GTC, generalized tonic clonic; IA, impaired awareness; M, myoclonic; MA, myoclonic atonic; PA, preserved awareness; TA, typical absence.

^a^ Normal w/ paucity of normal sleep architecture.

^b^ Episodes of status epilepticus controlled by valproic acid are consistent with Dravet syndrome, although FSIQ is higher than expected with no developmental delay.

^c^ Convulsive seizures controlled, atonic seizures persist.

**Table 2:** SCN1A variants and classifications.

Subject ID	Variant	Channel domain	Phenotype	Variant classification (clinical report)	De novo	Variant classification (post parental testing)	In vitro study results	In silico neuron firing (DB threshold)	Variant classification (after in vitro studies)	ACMG criteria
1	I1356M	D3/S5	G / F	VUS	Y	LP	Mixed	<	P^a^	PS2, PM1, PM2, PS3
2	L479P	ID1‐2	G / F	VUS	N^b^	LB	Partial LoF	< vs =	VUS^a^	PM2, BP4, BS4, PS3
3	R101W	N‐term	DS	P	Y	P	LoF	<<	P	PS2, PM1, PM2, PS3
4	K1737*	D4/pore loop	DS	P	–	P	–	<<	P	PSV1, PM2
5	C1588R	D4/S2	DS	LP	Y	P	LoF	<<	P^a^	PS2, PM1, PM2, PP3, PS3
6	M400del	D1/S6	DS	P	Y	P	LoF	<<	P	PS4, PS2, PM1, PS3
7	F403L	D1/S6	FS+	LP	–	LP	LoF	<<	P^a^	PS1, PM1, PM2, PP3, PS3
8	R393C	D1/pore loop	DS	P	–	P	LoF	<<	P	PM1, PM2, PP3
9	D1288A	D3/S3	DS	VUS	Y	LP	LoF	<<	P^a^	PS2, PM1, PM2, PP3
10	R542*	ID1‐2	FS+ –>DS	P	–	P	–	<<	P	PSV1
11	W748*	ID1‐2	DS	P	–	P	–	<<	P	PSV1, PM2
12	T1250M	D3/S1‐S2	EMA	VUS	N	VUS	WT	<<	LB^a^	PM1, BS3, BS4

Variant classification provided on initial clinical report, reclassification incorporating parental testing and final reclassification incorporating in vitro studies, along with ACMG criteria applied to determine the final classification. < indicates a decreased threshold for induction of depolarization block by a stimulus, << indicates a greatly decreased threshold for induction of depolarization block by a stimulus, and < vs = indicates decreased threshold for induction of depolarization block with select stimulus paradigms (EPSPs but not constant injected current).

DS, Dravet syndrome; EMA, epilepsy with myoclonic absences; FS+, febrile seizures+; FS+ −>DS, initially diagnosed as febrile seizures+, later diagnosed as Dravet; G/F, generalized and focal seizures, no specific syndrome; LB, likely benign; LP, likely pathogenic; P, pathogenic; VUS, variant of uncertain significance.

^a^ In vitro functional testing provided information that changed variant classification established after parental testing (if available).

^b^ Mother carries variant but has no history of epilepsy, father had childhood epilepsy but does not have variant.

### Functional assessment of SCN1A variants

We used automated patch clamp recording to determine the functional properties of nine non‐truncating *SCN1A* variants identified in the cohort. Six of the nine variants failed to produce sodium currents distinguishable from background currents in HEK293T cells (R101W, R393C, M400del, F403L, D1288A, and C1588R) (Fig. 2B,C). The other three variants produced currents large enough for further functional evaluation, and two of these variants (L479P, I1356M) exhibited abnormal functional properties (Table S2). While whole‐cell current density for L479P was similar to that of WT channels, the time‐constant for onset of inactivation was faster, and ramp and persistent currents were smaller, consistent with partial LoF. Currents mediated by I1356M were slightly smaller than WT channels and were more sensitive to frequency‐dependent channel rundown. However, this variant also exhibited larger window current compared to WT channels, suggesting that I1356M has a mix of LoF and GoF properties. Lastly, the biophysical properties of T1250M were indistinguishable from WT, suggesting that this variant is likely benign (Table S2).

### Neuronal action potential simulations

**Figure 3:**
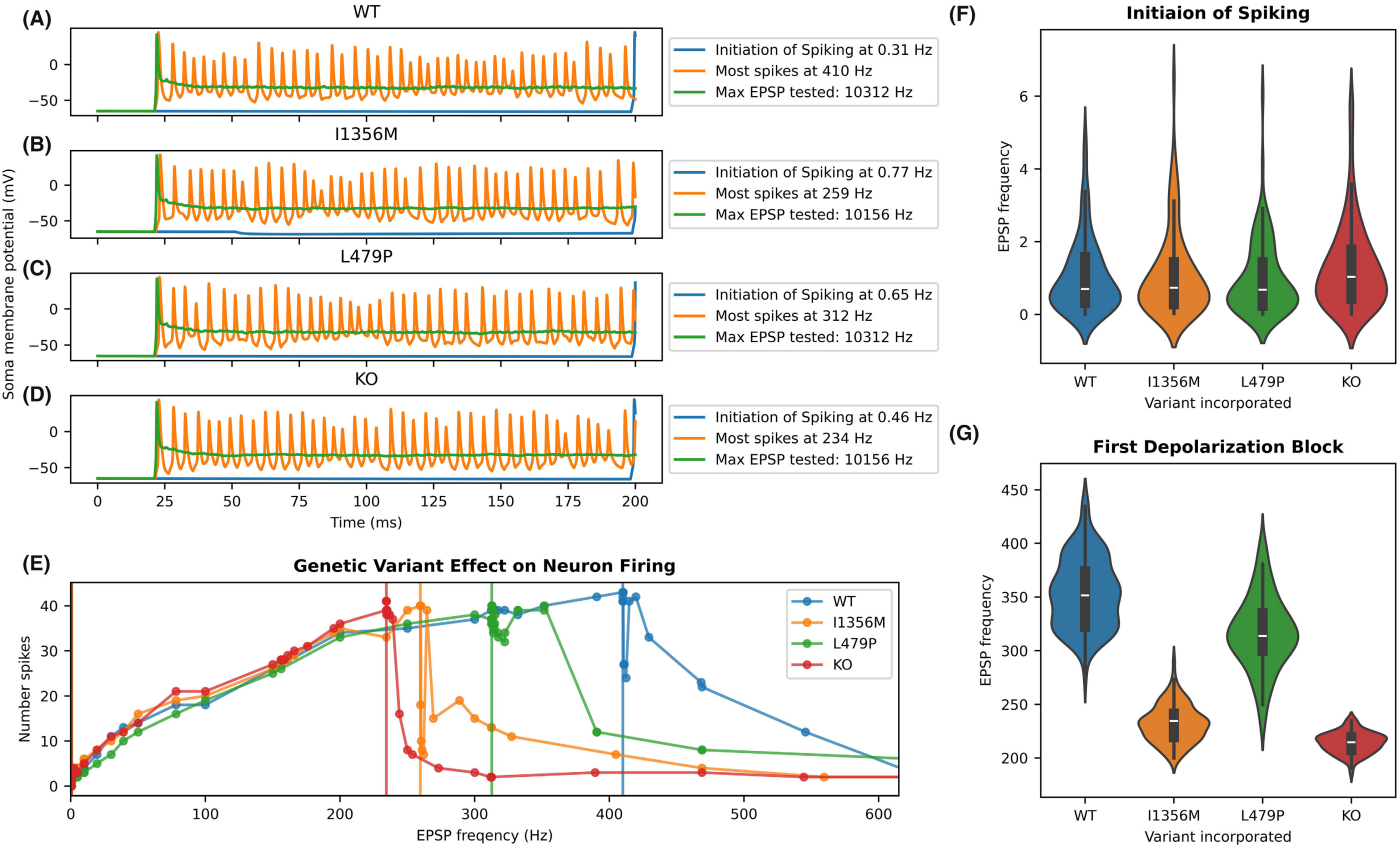
Stimulus frequency leading to first spike and depolarization block for stimulation at the soma of model neurons with wild type and variant channels. (A–D) representative plots of membrane potential for model neurons containing a heterozygous representation of each SCN1A variant type with stimulus yielding initial spike, largest number of neuronal spikes and at maximal frequency tested (providing a clear visualization of depolarization block). (E) Plot of number of neuron spikes for each stimulation frequency tested in different variants for a representative simulation run. Four lines indicating initial spike are present (but overlap) in the leftmost portion of the graph, lines indicating the point of maximal spikes that precedes transition to depolarization block are seen in the middle of the plot. (F) Violin plot of EPSP frequency yielding first spike in 100 simulation runs, showing no difference between variants. (G) Violin plots showing EPSP frequency yielding transition to depolarization block in 100 simulation runs, with the KO (knock‐out/total loss of function) variants first transitioning to depolarization block, then I1356M, then L479P, then WT.

To predict how Na_V_1.1 dysfunction affects interneuron firing, we incorporated in vitro functional properties for each variant into an anatomically detailed, biophysically based PV^+^‐interneuron computer model. We used a paradigm of serial simulations (see Methods) that compared firing frequency for wild type (WT) and heterozygous variant neurons across a range of stimulus intensities. This paradigm defined the stimulus intensity required for initiation of spiking and for subsequent failure of spiking (apparent depolarization block) for each variant. Examples of simulation output for different variants and stimulus intensity is shown in Figure 3A–D, and the relationship between stimulus intensity and simulated neuron action potential frequency is shown in Figure 3E. In a first run of 66 simulations, the stimulus was a fixed current injected at the soma. Rheobase was similar for all neurons: 4.6 pA for WT, L479P, and heterozygous knock out (KO), which describes seven variants with total LoF, and 4.8 pA for the I1356M variant. Above this threshold, all variant model neurons generated similar numbers of spikes for a given injected current up to the point of apparent depolarization block. In contrast, apparent depolarization block occurred at substantially lower injected current amplitudes for complete LoF variants (0.2 nA) and I1356M, (0.3 nA) compared to WT and L479P variants (0.4 nA).

In a second set of simulations, neurons were stimulated at the soma with a mixture of EPSPs and IPSPs. We conducted 100 simulation runs (79 simulations per run) with an EPSP:IPSP ratio of approximately 3:1. In these runs, PV^+^ interneuron firing frequency increased monotonically with increasing PSP frequency up to the point of apparent depolarization block (Fig. 3E). There were no differences among variant models in the frequency of PSPs that yielded initial spiking (Kruskal–Wallis ANOVA, *p* = 0.13, Fig. 3F) or in the number of action potentials produced at higher frequency PSP stimulation up to the point of apparent depolarization block (Fig. 3E). As in the first set of simulations, variant neuron models exhibited apparent depolarization block at lower stimulus intensity than WT neuron models (Kruskal‐Wallis ANOVA *p* = 3.36 × 10^−69^, Figure 3G). In contrast with simulations using injected current, the L479P variant had onset of apparent depolarization block at a lower EPSP frequency than WT (*p* < 0.001, Fig. 3G). These findings suggest a damaging effect of the L479P variant under select circumstances and that this variant may act a genetic epilepsy modifier rather than a primary cause of epilepsy.

### Genotype‐function‐phenotype correlations

Nine individuals were heterozygous for variants that caused complete LoF determined by patch clamp recording. Of these individuals, seven carried a diagnosis of Dravet syndrome, one (individual 7, F403L variant) carried a diagnosis of GEFS+, and one was initially diagnosed with GEFS+ but was later reclassified as Dravet syndrome (individual 10, R542*) (Table 1). Individual 7 had seizures easily controlled with valproic acid, and no developmental delay was noted between age 9 months and 2.5 years. Individual 6 (M400del variant) carried a diagnosis of Dravet syndrome but had a milder phenotype, characterized by prolonged focal seizures well‐controlled with valproic acid and cannabidiol, full scale IQ of 85 and strong school performance. All individuals with complete LoF variants had onset of seizures within the first year of life, with an average age of 5.8 months (IQR 3.6–8.1 months) and fevers as a seizure trigger. Phenotypes were otherwise heterogenous; seven (78%) had both focal and generalized seizures, four (44%) had myoclonic seizures, seven (78%) had prolonged seizures, and four (44%) had episodes of status epilepticus. EEG findings were also heterogenous, with seven individuals (78%) having at least one normal and one abnormal EEG. Five individuals with complete LoF variants had formal neuropsychology testing; FSIQ scores ranged from 40 to 85, with half of the individuals having FSIQ > 75 between 3 and 5 years of age. No individuals in this study had a history of developmental regression. Three of four individuals with complete LoF variants who did not have neuropsychology testing had developmental delay in the first year of life.

Three subjects did not have a phenotype in keeping with a Dravet syndrome/GEFS+ spectrum disorder. Two individuals who were heterozygous for partial LoF variants (I1356M, L497P) did not meet criteria for a specific epilepsy syndrome. Neither individual had febrile seizures, and onset of seizures was much later (3 and 5 years of life) compared to individuals with complete LoF variants. Neither individual with a partial LoF variant exhibited developmental delay; one had a FSIQ of 107, the other did not have neuropsychology testing. The individual carrying the benign T1250M variant had myoclonic epilepsy, attributed to a pathogenic *TBCK* variant identified after *SCN1A* testing.

**Figure 4:**
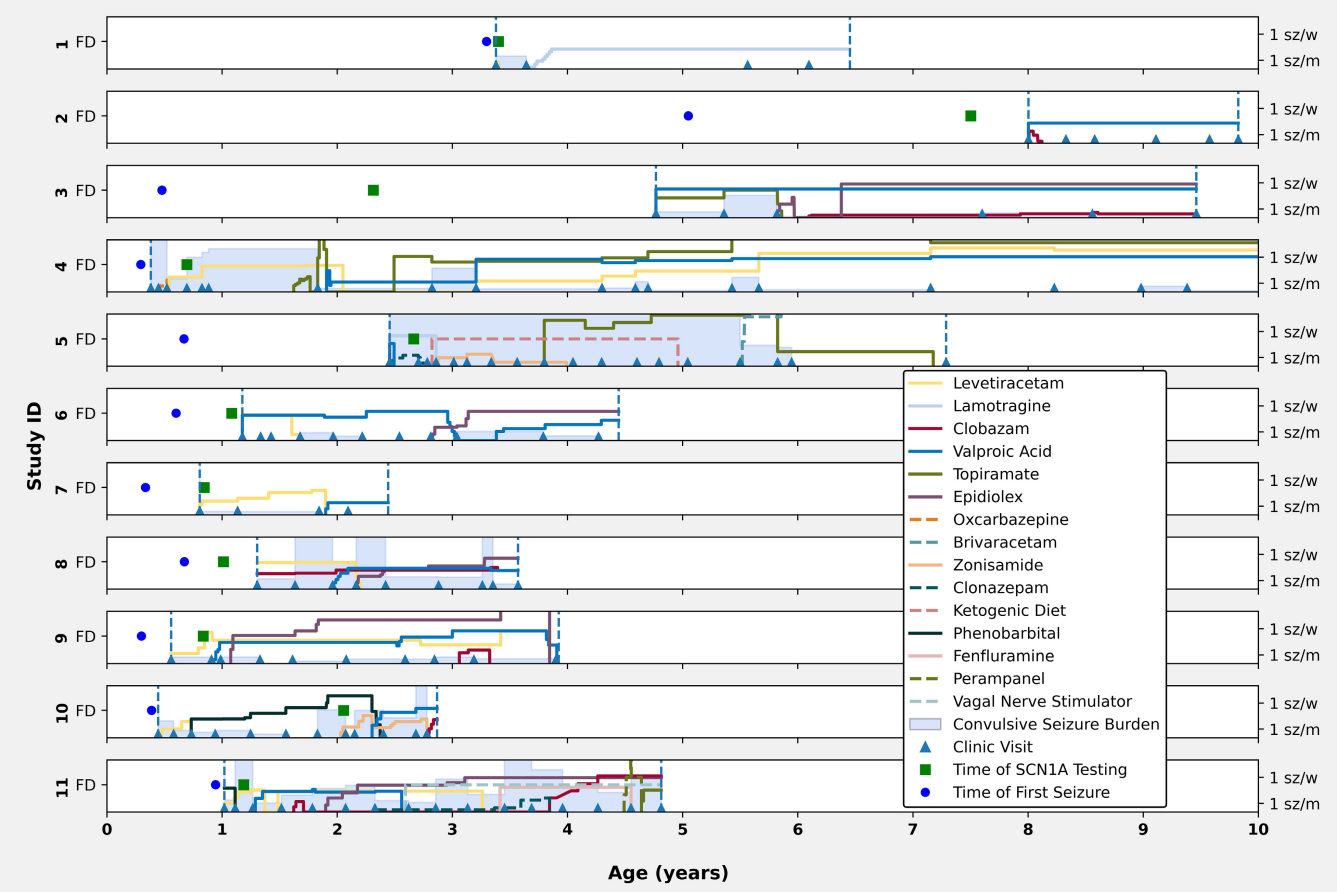
Clinical trajectory, seizure frequency over time and medication response for patients with SCN1A variants that were potentially pathogenic without another clear cause of epilepsy. Subjects were followed in clinic between the ages indicated by the dotted blue lines, with specific clinic visits indicated by small blue triangles. Seizure frequency between two clinic visits is indicated by the shaded blue area between two triangles (scale is seen on right, 1 sz/w – one seizure per week, 1 sz/m – one seizure per month). Medication dose is indicated by solid lines, with “FD” (Full Dose) indicating a typical maximal dose used for that medication (see methods). Age at seizure onset and SCN1A testing are indicated by blue circles and green squares respectively.

Individuals with variants affecting Na_V_1.1 function were followed for an average duration of 3.6 years (IQR 2.9–4.7 years). Over the course of the observation period, participants were treated with a spectrum of ASMs, with normalized weight‐based dosing over time illustrated in Figure 4. Four of nine individuals (44%) with complete LoF variants achieved freedom from sustained convulsive seizures during their last clinical follow up interval, and one additional individual had rare seizures (twice yearly). Valproic acid was trialed in all individuals with complete LoF variants and was associated with seizure control in four of nine individuals. One individual with a partial LoF variant also responded well to valproic acid. This drug was stopped due to side effects in three individuals. Individual 1 achieved complete seizure control with a low dose of lamotrigine, a medication often considered contraindicated for *SCN1A* LoF variants. No single ASM or ASM combination was universally effective.

### Reclassification of variants

Classifications of variants are provided in Table 2. Based upon initial clinical genetic reports, which rely on ACGM criteria, six variants were designated as pathogenic, two as likely pathogenic, and four as VUS. Parents of 7 of 12 individuals had genetic testing, leading to reclassification of four variants based on the evidence of de novo occurrence. After parental testing, the L479P variant was reclassified as likely benign because it was inherited from the mother who had no history of epilepsy and was not carried by the father who had childhood epilepsy. After applying ACMG criteria to results of in vitro and in silico studies, five variants were reclassified, three from likely pathogenic to pathogenic (C1588R, F403L, D1288A), one from VUS to likely benign (T1250M), and one from likely benign to VUS (L479P).

### Comparison to in silico prediction tools

**Figure 5:**
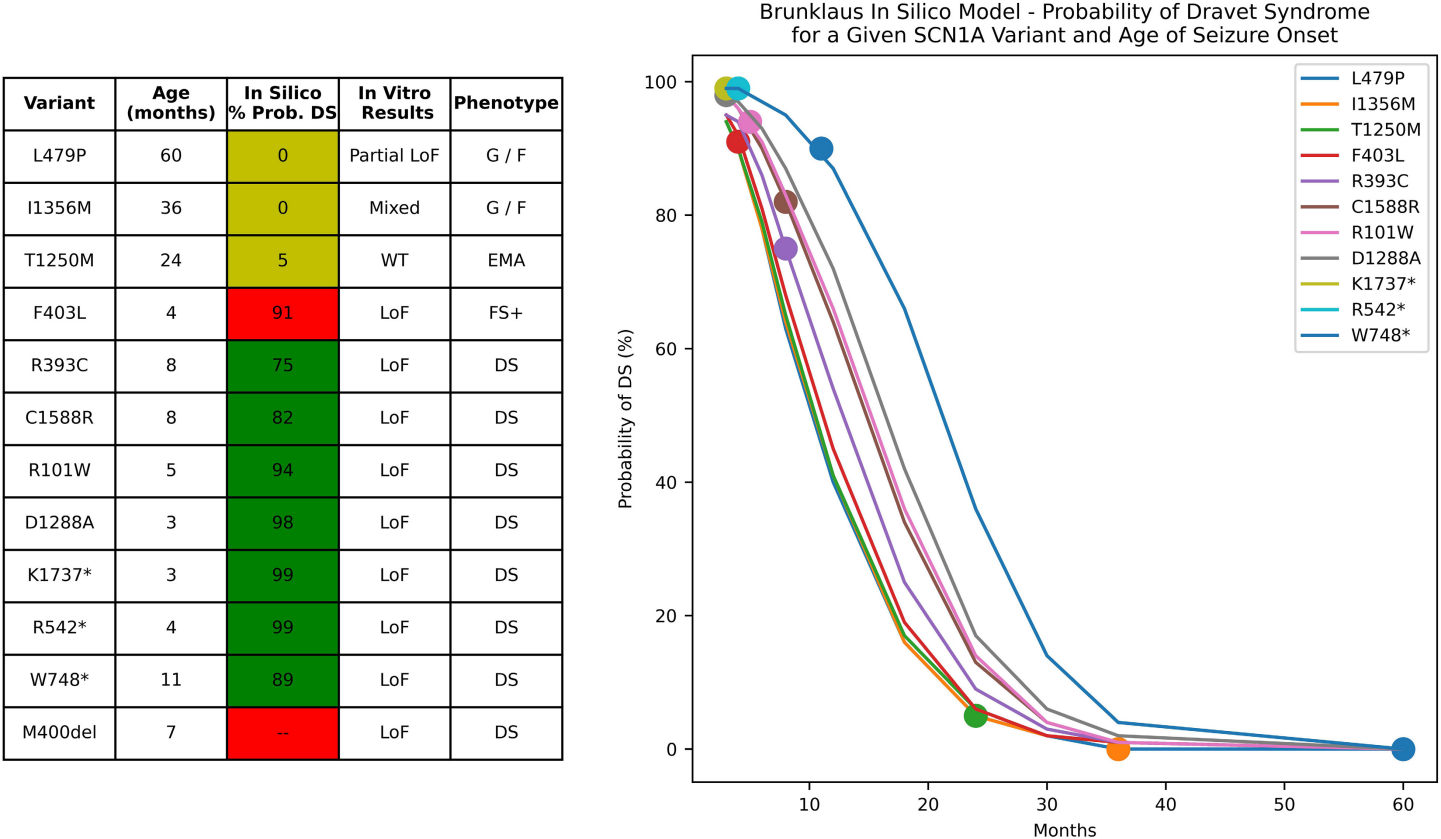
Comparison of phenotypic predictions (Dravet syndrome vs. genetic epilepsy with febrile seizures+) of an in silico model developed by Brunklaus^22^ with results of in vitro studies and observed patient phenotype. Correct predictions of Dravet syndrome are highlighted in green. One incorrect prediction (variant F403L) and one variant for which the in silico model could not generate a prediction (M400del) are highlighted in red. Three correct predictions of low probability of Dravet syndrome are highlighted in yellow. Although these predictions are correct, the in silico model does not distinguish between distinct non‐Dravet phenotypes or distinct changes in channel function that have significant clinical implications. DS, Dravet syndrome; EMA, epilepsy with myoclonic‐atonic seizures; G/F, generalized and focal seizure without febrile seizures or a specific epilepsy syndrome; LoF, loss of function; WT, wild type.

A publicly available prediction model^19^ correctly predicted a DS phenotype for 7 of 12 individuals (Fig. 5). This model incorrectly predicted the individual with F403L variant (which causes total LoF) and febrile seizures as having a 91% chance of developing Dravet syndrome and could not generate a prediction for in‐frame deletion (M400del). Age was a major driver of probability of Dravet syndrome, with all variants yielding a probability of 90% or greater at 4 months old, and all variants yielding a probability <15% at 30 months old. At a given age for seizure onset, the in silico model predicted the same probability of Dravet syndrome for the L479P, I1356M, T1250M, and F403L variants, even though these led to partial LoF, mixed LoF/GoF, no change in function and total LoF respectively, and did not distinguish between distinct clinical phenotypes.

## Discussion

This study of a representative sample of individuals with epilepsy and *SCN1A* variants demonstrates that genotype and channel function correlate with multiple aspects of the epilepsy phenotype. Seventy‐five percent of variants in this cohort caused complete LoF, which were universally associated with seizure onset before one year of age and febrile seizures. This is consistent with larger clinical studies showing that Dravet syndrome is strongly correlated with onset of seizures in the first year of life.^26^, ^27^ Complete LoF variants were generally associated with worse than average cognitive outcomes, and were generally associated with drug resistant epilepsy. In contrast, 17% of variants led to partial LoF or mixed LoF/GoF. These variants were associated with seizure onset at 3–5 years of age, no febrile seizures, apparently normal cognition, and medication‐responsive epilepsy.

Cognitive outcomes and drug‐resistant epilepsy were not universally predicted by channel function; individuals with complete LoF variants had substantial phenotypic variability. One individual with a complete LoF variant (F403L) had medication‐responsive epilepsy and no apparent developmental delay. Furthermore, other phenotypic features including seizure type, seizure duration, EEG findings, and seizure control were variable in participants with complete LoF variants. Cognitive measures also varied between individuals with complete LoF variants. However, this apparent cognitive variability could be confounded by age at time of neuropsychology testing – children with Dravet syndrome often have slower development of cognitive function relative to the general population in the later portion of childhood.^28^

Variability in clinical phenotypes and outcomes could stem in part from different effects of specific variants on channel expression, trafficking and localization, which are not captured by electrophysiological recordings. Additionally, individuals with complete LoF variants and milder phenotypes may have more effective homeostatic mechanisms that compensate for lower maximal Na_V_1.1 current.^29^, ^30^, ^31^ Furthermore, other factors such as genetic modifiers and developmental network properties likely modulate epilepsy phenotype. Although clinical genetic testing did not report somatic mosaicism for *SCN1A* variants, undetected mosaicism remains a potential cause for milder phenotypes.

In contrast to other in silico prediction models that use genetic and clinical data^32^, ^33^ or phenotype,^19^ our study uses in silico biophysical neuron modeling to predict how channel dysfunction affect neuron firing. Such modeling is a useful adjunct for assessing potential neurophysiological effects of partial LoF, GoF, or mixed LoF/GoF variants. For example, the L479P variant is unlikely to be the primary cause of epilepsy in individual 2, as this variant is carried by the individual's mother who did not have epilepsy, whereas her father who had a similar childhood epilepsy did not carry this variant. However, in vitro studies show that this variant causes faster onset of inactivation as well as smaller persistent current. Neuron simulations confirmed that these changes are expected to cause a lower threshold for apparent depolarization block (as has been described for SCN1A GoF variants^17^) but only with some paradigms. Thus, neuron modeling helps confirm that in vitro results are expected to affect neuron firing, which in turn leads to reclassification of the L479P from likely benign to VUS using ACMG criteria, raising the possibility that this variant may be a modifier of the epilepsy phenotype. The publicly available SCN1A predictive model tested in these studies did not distinguish between the L479P, I1356M, T1250M, and F403L variants, even though these led to partial LoF, mixed LoF/GoF, no change in function and total LoF respectively. Although the in silico model's phenotypic prediction (no Dravet syndrome) was correct for three of these four individuals when taking their age into account, distinguishing channel dysfunction between these variants is clinically important, both for diagnosis and medical management. For this set of 12 variants, electrophysiological assessment and neurophysiological modeling led to reclassification of five variants using ACMG criteria, highlighting the potential clinical value of this approach.

Our results highlight several clinical considerations for medical management of children with epilepsy due to *SCN1A* variants. In keeping with larger clinical studies,^19^, ^26^, ^27^, ^34^ we found age of seizure onset to be most predictive of development of a Dravet syndrome phenotype. A recent ILAE position statement defines mandatory criteria for diagnosis of Dravet syndrome as onset of febrile and afebrile recurrent focal and/or generalized seizure between 1 and 20 months and drug‐resistant epilepsy with intellectual disability.^35^ However, drug resistance and intellectual disability are often not apparent until months to years after seizure onset, yet individuals with complete LoF variants would benefit from targeted treatments for Dravet syndrome at time of seizure onset. Following valproic acid (the recommended first line ASM for Dravet syndrome which was also best associated with seizure freedom in this cohort), recommended medications such as fenfluramine or cannabidiol require a specific diagnosis of Dravet syndrome for insurance approval. Thus, given the high prevalence of total LoF variants and phenotypic variability seen in this study, the clinician should consider use of the diagnosis of Dravet syndrome for patients with complete LOF SCN1A variants presenting with febrile seizures before one year of age, even if they have not yet developed drug‐resistant epilepsy or intellectual disability.^7^, ^36^

In conclusion, our results demonstrate that automated patch clamp recording has value for resolving uncertainty in *SCN1A* variant classification and offers additional insight into genotype‐function‐phenotype correlations including medication responses for a cohort of individuals with *SCN1A*‐related disorders. Use of neuron simulation studies to predict the impact of channel dysfunction on PV+ neuron firing provides additional value in defining neurophysiological mechanisms underlying these disorders. Extending these paradigms to simulate networks of neurons and medication effects^37^ could further precision medicine paradigms to better understand pathogenicity of genetic variants and predict effective treatments for patients with genetic epilepsy.

## Author Contributions

It should read: A.T.K. and A.L.G. conceived and planned the experiments. C.H.T. and T.V.A. carried out high‐throughput patch clamp experiments, and C.H.T. did analysis of in vitro data. A.T.K. developed the computer model used, conducted simulations; A.T.K., B.S., and D.S. analyzed simulation results. A.T.K. and A.F. extracted and analyzed clinical data. A.T.K. wrote the manuscript, with iterative feedback from A.L.G. and C.H.T. All authors provided critical feedback and helped shape the research, analysis and manuscript.

## Conflict of Interest

The authors have no relevant conflicts of interest to disclose.

## Supporting information


**Dataset S1.** Biophysical properties of *SCN1A* variants.

**Table S1.** Mutagenic *SCN1A* primers.
**Table S2.** Functional properties of *SCN1A* variants.

## Data Availability

The data that supports the in vitro findings of this study are available in the supporting information of this article. The neuron model used may be found at modelDB at the URL listed in the manuscript.
