# Supplementary material for: Genotype‐function‐phenotype correlations for SCN1A variants identified by clinical genetic testing
Source: Ann Clin Transl Neurol. 2025 Jan 21;12(3):499–511. doi: 10.1002/acn3.52297 (PMC11920720; doi:10.1002/acn3.52297)
Supplement: Supplementary file 2 — Table S1. Mutagenic SCN1A primers. Table S2. Functional properties of SCN1A variants. [file ACN3-12-499-s001.docx]

**Genotype-Function-Phenotype Correlations for *SCN1A* Variants Identified by Clinical Genetic Testing**

Andrew Knox, M.D. M.S., Christopher H. Thompson, Ph.D., Dillon Scott, B.S., Nora F. Ghabra, B.S., Tatiana V. Abramova, M.S., Bethany Stieve, Ph.D., Abi Freeman, M.S., and Alfred L. George, Jr., M.D.

**SUPPLEMENTAL INFORMATION**

**Supplemental Tables**

**Table S1.** Mutagenic *SCN1A* primers.

**Table S2.** Functional properties of *SCN1A* variants

**Supplemental Datasets**

**Dataset 1.** Biophysical properties of *SCN1A* variants

|  |  | |  | |
| --- | --- | --- | --- | --- |
| **Variant** | **Forward Primer Sequence** | **Reverse Primer Sequence** | |  |
| **R101W** | AGGGAAGGCCATCTTCTGGTTCAGTGCCACCTCTGCCC | ACCAGAAGATGGCCTTCCCTTTATTCAATACTATAAAAG | |  |
| **R393C** | AACTGACATTATGTGCTGCTGGGAAAACGTACATGATATTC | GCAGCACATAATGTCAGTTGATAAAGATTTTCCCAGAAGTCC | |  |
| **M400del** | GTACAAAATATTCTTCGTATTGGTCATTTTCTTGGGCTCAT | ATACGAAGAATATTTTGTACGTTTTCCCAGCAGCACGTAATG | |  |
| **F403L** | TGATATTCCTCGTATTGGTCATTTTCTTGGGCTCATTCTAC | GACCAATACGAGGAATATCATGTACGTTTTCCCAGCAGCAC | |  |
| **L479P** | GCAGGCCCTCAGACAGCTCATCTGAAGCCTCTA | TGAGCTGTCTGAGGGCCTGCCTGCTGCACTGGG | |  |
| **T1250M** | AAGACGATTAAGATGATGTTGGAATATGCTGACAAGGTTTTCACTTAC | AACATCATCTTAATCGTCTTTCGCTGATCAATATATATATCTTCAAAT | |  |
| **D1288A** | GGCCTTCTTAATTGTTGATGTTTCATTGGTCAGTTTAACAG | CATCAACAATTAAGAAGGCCAGCCAACACCAGGCATTGGTG | |  |
| **I1356M** | GGTTTGTCTTATGTTCTGGCTAATTTTCAGCATCATGGGCG | GCCAGAACATAAGACAAACCAGAAGCACATTCATGATGGATG | |  |
| **C1588R** | TTACTGGAGAGCGTGTACTGAAACTCATCTCTCTACGCC | CAGTACACGCTCTCCAGTAAATAGCACAATGAACACCAG | |  |

| **Supplemental Table S1:** Mutagenic SCN1A Primers | | | | |
| --- | --- | --- | --- | --- |
|  |  |  |  |  |
|  |  |  |  |  |
|  |  |  |  |  |
| **Current Amplitude (pA/pF)** | **WT** | **L479P** | **T1250M** | **I1356M** |
|  |  |  |  |  |
| Mean ± 95% CI | -104.5 ± 8.2 | -103.9 ± 12.5 | -73.1 ± 11.1 | **-83.4 ± 13.6** |
| % WT ± 95% CI | 100.0 ± 7.2 | 94.4 ± 11.3 | 82.1 ± 12.5 | **59.7 ± 14.3** |
| n | 365 | 83 | 107 | **107** |
| p-value |  | >0.9999 | >0.9999 | **0.0150** |
|  |  |  |  |  |
| **V_1/2_ Activation (mV)** |  |  |  |  |
|  |  |  |  |  |
| Mean ± 95% CI | -3.7 ± 0.6 | -5.4 ± 1.1 | -4.3 ± 1.4 | **-5.7 ± 0.9** |
| Δ WT ± 95% CI | 0.0 ± 0.5 | 0.7 ± 1.1 | -1.5 ± 1.4 | **-3.9 ± 0.9** |
| n | 191 | 44 | 36 | **60** |
| p-value |  | 0.5656 | 0.8726 | **<0.0001** |
|  |  |  |  |  |
| **V_1/2_ Inactivation (mV)** |  |  |  |  |
|  |  |  |  |  |
| Mean ± 95% CI | -36.7 ± 0.8 | -38.9 ± 1.3 | -34.5 ± 1.5 | -34.7 ± 1.5 |
| Δ WT ± 95% CI | 0.0 ± 0.8 | -0.2 ± 1.3 | -0.8 ± 1.4 | 1.4 ± 1.5 |
| n | 308 | 53 | 71 | 69 |
| p-value |  | 0.5731 | 0.9391 | 0.0881 |
|  |  |  |  |  |
| **Recovery Tau Fast (ms)** |  |  |  |  |
|  |  |  |  |  |
| Mean ± 95% CI | 4.8 ± 1.1 | 4.1 ± 1.5 | 3.7 ± 1.3 | 8.5 ± 2.7 |
| Fold WT ± 95% CI | 1.0 ± 0.3 | 1.1 ± 0.4 | 0.9 ± 0.1 | 1.4 ± 0.4 |
| n | 90 | 61 | 17 | 40 |
| p-value |  | 0.2260 | 0.7101 | 0.5989 |
|  |  |  |  |  |
| **Recovery Tau Slow (ms)** |  |  |  |  |
|  |  |  |  |  |
| Mean ± 95% CI | 102.7 ± 17.2 | 87.1 ± 11.2 | 103.4 ± 33.9 | 108.2 ± 17.4 |
| Fold WT ± 95% CI | 1.0 ± 0.2 | 0.8 ± 0.1 | 1.1 ± 0.3 | 1.0 ± 0.2 |
| n | 90 | 61 | 17 | 40 |
| p-value |  | 0.9888 | 0.7277 | >0.9999 |
|  |  |  |  |  |
| **Recovery % Fast** |  |  |  |  |
|  |  |  |  |  |
| Mean ± 95% CI | 53.5 ± 1.9 | 56.8 ± 3.2 | **62.4 ± 4.2** | **45.5 ± 4.2** |
| % WT ± 95% CI | 100.0 ± 3.7 | 104.6 ± 5.8 | **121.9 ± 8.2** | **84.9 ± 7.9** |
| n | 90 | 61 | **17** | **40** |
| p-value |  | 0.2220 | **0.0012** | **0.0021** |
|  |  |  |  |  |
| **Use-Dependent Rundown (P_30_/P_1_)** |  |  |  |  |
|  |  |  |  |  |
| Mean ± 95% CI | 91.6 ± 0.7 | 91.0 ± 0.2 | 92.9 ± 1.4 | **88.3 ± 0.2** |
| % WT ± 95% CI | 100.0 ± 0.7 | 97.7 ± 1.7 | 99.9 ± 1.5 | **96.3 ± 1.9** |
| n | 272 | 76 | 34 | **70** |
| p-value |  | 0.0650 | 0.9226 | **0.0099** |
|  |  |  |  |  |
| **Inactivation Tau (ms)** |  |  |  |  |
|  |  |  |  |  |
| Mean ± 95% CI | 1.2 ± 0.5 | **1.2 ± 0.1** | 1.3 ± 0.1 | 1.2 ± 0.1 |
| % WT ± 95% CI | 100.0 ± 4.5 | **92.7 ± 6.7** | 109.8 ± 9.8 | 98.8 ± 6.2 |
| n | 319 | **89** | 71 | 76 |
| p-value |  | **0.6755** | 0.2166 | >0.9999 |
|  |  |  |  |  |
| **Ramp Current (pC/nA)** |  |  |  |  |
|  |  |  |  |  |
| Mean ± 95% CI | 6.3 ± 0.7 | **5.6 ± 0.9** | 5.3 ± 0.8 | 6.2 ± 1.0 |
| % WT ± 95% CI | 100.0 ± 10.0 | **72.1 ± 11.0** | 98.4 ± 14.9 | 119.3 ± 19.5 |
| n | 100 | **28** | 26 | 19 |
| p-value |  | **0.0107** | 0.7722 | 0.0705 |
|  |  |  |  |  |
| **Persistent Current (% Peak)** |  |  |  |  |
|  |  |  |  |  |
| Mean ± 95% CI | 3.6 ± 0.7 | **3.0 ± 0.6** | 2.9 ± 0.7 | 2.9 ± 0.5 |
| % WT ± 95% CI | 100.0 ± 13.8 | **70.9 ± 14.8** | 106.8 ± 23.4 | 104.5 ± 19.4 |
| n | 100 | **28** | 11 | 19 |
| p-value |  | **0.0192** | 0.6697 | 0.3640 |
|  |  |  |  |  |
| **Window Current (Area)** |  |  |  |  |
|  |  |  |  |  |
| Mean ± 95% CI | 0.5 ± 0.1 | **0.5 ± 0.1** | 0.6 ± 0.1 | **0.9 ± 0.2** |
| % WT ± 95% CI | 100.0 ± 9.6 | **83.9 ± 12.9** | 125.1 ± 22.6 | **196.0 ± 44.9** |
| n | 113 | **48** | 30 | **28** |
| p-value |  | **0.0239** | 0.0658 | **0.0013** |

**Supplemental Table S2:** Functional Properties of *SCN1A* Variants
